# Microarray: a global analysis of biomineralization-related gene expression profiles during larval development in the pearl oyster, *Pinctada fucata*

**DOI:** 10.1186/s12864-015-1524-2

**Published:** 2015-04-19

**Authors:** Jun Liu, Dong Yang, Shiting Liu, Shiguo Li, Guangrui Xu, Guilan Zheng, Liping Xie, Rongqing Zhang

**Affiliations:** Institute of Marine Biotechnology, Collaborative Innovation Center of Deep Sea Biology, School of Life Science, Tsinghua University, Beijing, 100084 China

**Keywords:** Microarray, Larval development, Shell formation, Matrix proteins, Biomineralization, *Pinctada fucata*

## Abstract

**Background:**

The molluscan *Pinctada fucata* is an important pearl-culturing organism to study biomineralization mechanisms. Several biomineralization-related genes play important roles regulating shell formation, but most previous work has focused only on their functions in adult oysters. Few studies have investigated biomineralization during larval development, when the shell is initially constructed and formed until the juvenile stage in dissoconch shells. Here, we report, for the first time, a global gene analysis during larval development of *P. fucata* based on a microarray and reveal the relationships between biomineralization-related genes and the shell formation process.

**Results:**

Based on the *P. fucata* mantle transcriptome, 58,940 probes (60 nt), representing 58,623 transcripts, were synthesized. The gene expression profiles of the fertilized egg, trochophore, D-shaped, and umbonal stage larvae, as well as juveniles were analyzed by microarray performance. The expression patterns of the biomineralization-related genes changed corresponding to their regulatory function during shell formation. Matrix proteins chitin synthase and PFMG2 were highly expressed at the D-shaped stage, whereas PFMG6、PFMG8 and PfN23 were significantly up-regulated at the umbonal stage, indicating different roles regulating the formation of either periostracum, Prodissoconch I or Prodissoconch II shells. However, the majority of matrix proteins were expressed at high levels at the juvenile stage, and the shells comprised both an aragonitic nacreous layer and a calcitic prismatic layer as adults. We also identified five new genes that were significantly up-regulated in juveniles. These genes were expressed particularly in the mantle and coded for secreted proteins with tandem-arranged repeat units, as most matrix proteins. RNAi knockdown resulted in disrupted nacreous and prismatic shell layers, indicating their potential roles in shell formation.

**Conclusions:**

Our results add a global perspective on larval expression patterns of *P. fucata* genes and propose a mechanism of how biomineralization-related genes regulate the larval shell formation process. These results increase knowledge about biomineralization-related genes and highlight new aspects of shell formation mechanisms.

**Electronic supplementary material:**

The online version of this article (doi:10.1186/s12864-015-1524-2) contains supplementary material, which is available to authorized users.

## Background

The pearl oyster *Pinctada fucata* is one of the most economically important bivalves in the pearl industries of the Japan and South China Seas, and is also a good molluscan species to study biomineralization [1].

The shell of *P. fucata* comprises the inner aragonitic nacreous layer and the outer calcitic prismatic layer, both of which comprise calcium carbonate and small amounts of organic macromolecules, including proteins, polysaccharides, and lipids [2]. These macromolecules, particularly the matrix proteins, comprise < 5% of shell weight but play important roles in nucleation, polymorphism, orientation, morphology, and organization of the calcium carbonate crystallites during shell formation [3]. Several shell matrix proteins have been separated and reported to have special effects on one layer or both. For example, nacrein [4], MSI60 [5], pearlin [6], N19 [7], and Pif [8] play essential roles in the nacreous layer, whereas MSI31 [5], prismalin-14 [9], aspein [10], prisilkin-39 [11], and the KRMP family [12] participate in the prismatic layer; the shematrin family [13] is involved in both layers. Matrix proteins are secreted by the mantle tissue, which covers the inner surface of the shell [14-16]. This orientation allows the mantle tissue to play a key role in shell formation, as well as in pearl culture [17,18]. Although most matrix proteins are unique in structure and function, their primary structures are usually organized into different functional domains with tandem-arranged repeat units [14].

According to previous studies, six developmental stages have been described across the entire *P. fucata* life cycle, including descriptions of the fertilized egg, trochophore stage, D-shaped stage, umbonal stage, juvenile, and adult [19]. The calcium carbonate crystal polymorphisms and the shell layer structure change during these stages [19,20]. Prodissoconch I, which probably comprises amorphous calcium carbonate (ACC), forms at the early D-shaped stage, whereas Prodissoconch II, which comprises aragonite and calcite, appears in the late D-shaped and umbonal stages [21]. The dissoconch shell, with an inner aragonitic nacreous layer and an outer calcitic prismatic layer, forms at the juvenile stage and grows throughout life [22]. The expression levels of six matrix proteins (nacrein, N16, prismalin-14, aspein, MSI60, and MSI31) have been investigated and confirmed to be involved in larval shell formation [21]. Two additional matrix proteins, PfN23 and PfN44, play essential roles in the *P. fucata* larval shell formation process [23,24]. However, changes in gene expression levels during larval development are poorly understood, which limits deeper insight into the gene regulatory mechanisms of the larval developmental process, particularly the control of shell formation.

Marine bivalves have been investigated using genomics tools [25-27], and preliminary studies have been conducted on adult *P. fucata* [18,28,29]. We have previously sequenced and characterized the *P. fucata* transcriptome from mantle tissue, which is the most important tissue during shell and pearl formation, with 58,623 unigenes (unpublished data). We have synthesized probes based on these sequences and performed a microarray analyses to study the different developmental stages and related gene expression profiles in *P. fucata*. We analyzed the global gene expression profiles of the *P. fucata* fertilized egg, trochophore, D-shaped stage, umbonal stage, and juvenile stage. The results reveal that most genes involved in biomineralization, including nacrein, pearlin, Pif, ACCBP, prisilkin-39, and the shematrin family are highly up-regulated in juveniles. In addition, chitin synthase is up-regulated to a greater extent at the D-shaped stage and is then highly expressed later, whereas the tyrosine metabolic pathway is continuously active throughout the D-shaped, umbonal, and juvenile stages. We also identified five secreted proteins with tandem-arranged repeat units, which were up-regulated > 20-fold between the umbonal larval and juvenile. Four genes were expressed briefly in the parallel mantle, mantle edge, or both. RNAi knockdown of these genes resulted in different disordered structures either in the nacreous or prismatic shell layers, suggesting potential roles in the regulation of shell formation. Our results have described the temporal expression and relative levels of RNA accumulation during larval shell development, increased the understanding of the molecular mechanisms, and the knowledge of biomineralization-related genes.

## Results

### Global gene expression analysis

**Figure 1:**
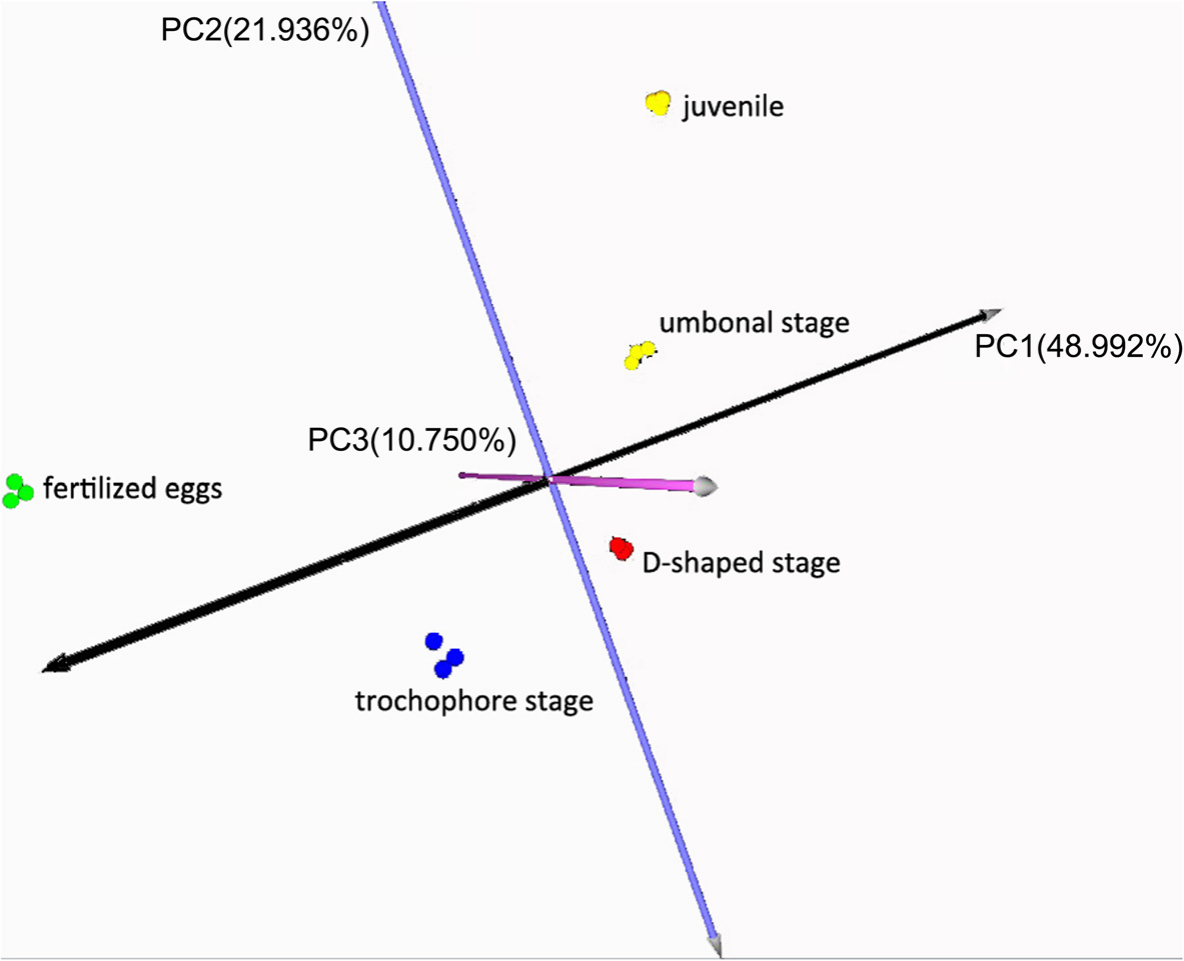
3D score plot using the first 3PCs identified by principal component analysis on the entire larval gene probe set. Fifteen pools of samples were separated into five groups, relevant to the five larval developmental stages. Each stage point included a triplicate.

The Raw and normalized fluorescence microarray data have been deposited in the GEO database under Accession Number GSE63824. A principal components analysis (PCA) on the entire probe set separated all 15 sample pools into five groups (Figure 1), which were relevant to the five larval developmental stages. The first and second components explained 70.93% of the variation in the entire data set. Stages of larval development was explained by the first principle components (PC1), which explained 48.99% of the variation, with low component loading for earlier stages and high component loading for later stages. A curved “horseshoe effect” along the second axis was apparent, which placed the trochophore and D-shaped stage larvae on the opposite side from fertilized eggs and the juvenile stage. Comparable results were obtained according to similarities in the expression patterns after a hierarchical clustering analysis (Additional file 1). These results indicate different gene expression patterns corresponding to different developmental stages. Meanwhile, the three biological replicate pools at each developmental stage shared a similar pattern, which would also increase the reliability and repeatability of this work.

### Transcriptional changes across larval stage transitions

**Figure 2:**
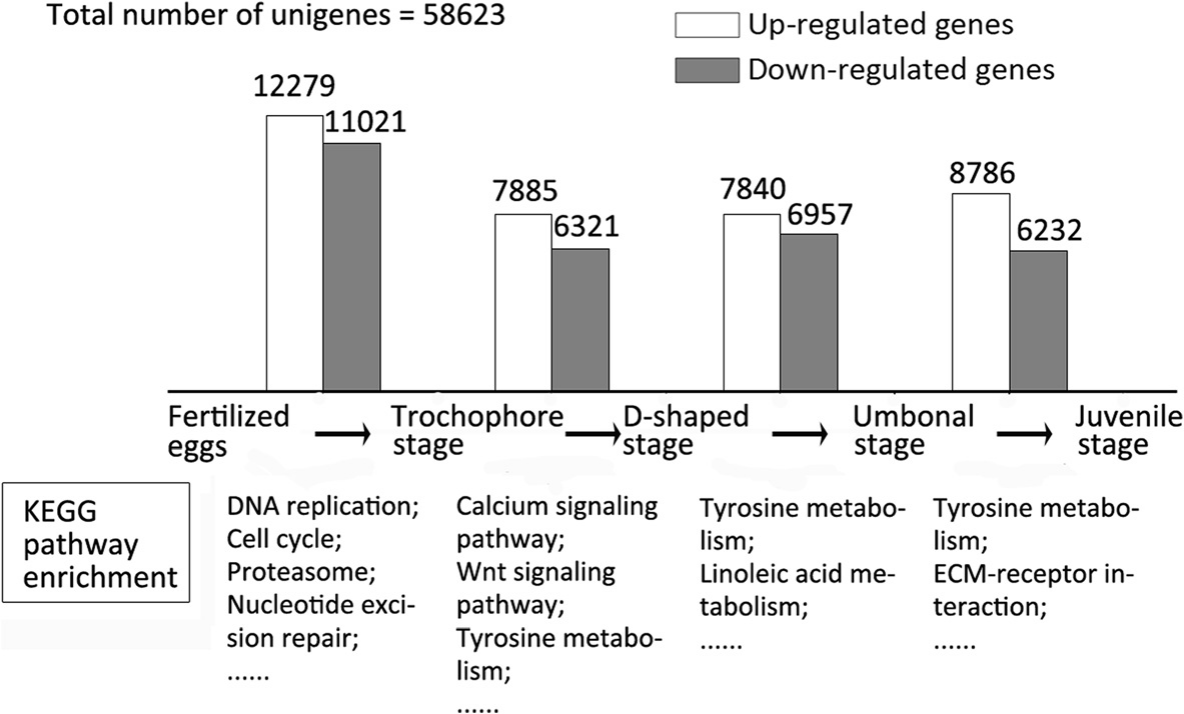
Transcriptional changes across larval stage transitions. The up-regulated genes refer to those with FC ≥ 2 while the down-regulated genes refer to those with FC ≤ 0.5; KEGG pathway enrichment shows several of those significantly enriched between two consecutive stages (q-value < 0.05), while the whole results could be seen in Additional file 3.

A comparative analysis was performed using SAM software to identify changes in expression profiles between two consecutive developmental stages [30]. Among all 58,749 unigenes, the greatest number of differentially expressed genes was found between fertilized eggs and trochophore stage larvae, with 23,300 significant genes (12,279 up-regulated and 11,021 down-regulated). However, the fewest genes changed between the D-shaped stage and trochophore stage, with 14,197 significant genes (7,885 up-regulated and 6,312 down-regulated). The expression profiles of most genes changed significantly during larval development (Figure 2). Cellular Component Gen Ontology (GO) (Additional file 2) and Kyoto Encyclopedia of Genes and Genomes (KEGG) pathway (Additional file 3) terms were represented significantly (q-value < 0.05) between the two consecutive stages.

### Comparison of fertilized eggs and trochophore stage larvae

Seventeen KEGG pathways (q-value < 0.05) were significantly enriched between fertilized eggs and trochophore stage larvae. The majority was related to cellular progression (e.g., ko03030://DNA replication, ko04110://cell cycle, ko03420://nucleotide excision repair, ko03430://mismatch repair, ko03440://homologous recombination). The genes that changed significantly between these two stages were related to GO:0005634//nuclear and GO:0005622//intracellular processes in a Cellular Components GO term analysis.

### Comparison of trochophore stage and D-shaped stage larvae

As many as 31 KEGG pathways (q-value < 0.05) were significantly enriched between the trochophore and D-shaped stages. Three pathways related to biomineralization and shell formation were enriched, such as (ko04020://calcium signaling pathway, ko04310://Wnt signaling pathway, and ko00350://tyrosine metabolism. Chitin synthase [15] was highly up-regulated with fold-change (FC) values of: CUST_58227, 84.49-fold increase; CUST_24765, 75.81-fold increase; CUST_39082, 60.81-fold increase; and CUST_56685, 34.93-fold increase, indicating their potential roles controlling shell formation, as chitinous material is synthesized during the D-shaped stage and contributes to the shell framework. Matrix protein PFMG2 [31] (CUST_40154, 5.25-fold increase) was also up-regulated at this stage and is probably involved in the formation of Prodissoconch I.

### Comparison of D-shaped stage and umbonal stage larvae

Only 10 KEGG pathways (q-value < 0.05) were significantly enriched when D-shaped and umbonal stage larvae were compared. Most of the terms were related to metabolic processes (e.g. ko00982://drug metabolism-cytochrome P450, ko00591://linoleic acid metabolism, ko00480://glutathione metabolism ko00260://glycine, serine, and threonine metabolism). Interestingly, the tyrosine metabolic pathway was also involved in these stages. PFMG6 and PFMG8 [31] were initially up-regulated at the D-shaped stage and then up-regulated to a greater extent at the umbonal stage.

### Comparison of umbonal stage larvae and juveniles

A total of 20 KEGG pathways (q-value < 0.05) were significantly enriched between umbonal stage larvae and juveniles, and protein members in tyrosine metabolic pathway showed the greatest increased. A Cellular Component GO analysis identified 16 terms for these significantly changed genes. The terms “extracellular region” and “extracellular matrix” were related to matrix proteins. As expected, most known matrix proteins were up-regulated to a greater extent at the juvenile stage, suggesting their particular roles regulating formation of either the inner aragonitic nacreous layer or the outer calcitic prismatic layer.

### Expression of the tyrosine metabolic pathway from the trochophore stage to juveniles

Of all of the KEGG pathways predicted to be involved in the comparison between two consecutive developmental stages, the tyrosine metabolic pathway (ko00350) was continuously active from the trochophore stage to juveniles with a q-value < 0.05. Among all 202 contigs from the transcriptome involved in this pathway, 70 were up or down-regulated from the trochophore stage to the D-shaped stage, 79 were up or down-regulated from the D-shaped stage to the umbonal stage, and 96 were up or down-regulated from the umbonal stage to juveniles (Additional file 3). Twenty-six genes were involved in all three comparisons (Table 1). The most significantly expressed gene was tyrosinase (CUST_54654), which was 716.82-fold up-regulated from the umbonal stage to juveniles, as well as two other tyrosinase-like proteins (tyrosinase-like protein 1, CUST_11152 and tyrosinase-like protein 2, CUST_41072) (Table 2). A previous study reported that tyrosinase is located in the prismatic layer of the shell [32] and is expressed particularly in the outer epithelial cells of the mantle middle fold, where periostracum formation occurs [33]. In addition to tyrosinase, melanin, the final metabolic product, is also thought to play a role in cuticle sclerotization in insects. Taken together, these results suggest multiple functions of the tyrosine metabolic pathway during *P. fucata* larval development, including shell and periostracum formation.

**Table 1:** **Members of the tyrosine metabolic pathway up or down-regulated consecutively between the trochophore larval stage and juveniles**

**Probe name**	**Unigene name**	**Homologous gene**	**Species**
CUST_54654	Unigene54694	Tyrosinase	*Pinctada fucata*
CUST_58138	Unigene58178	astacin-like protein	*Pinctada fucata*
CUST_6716	Unigene6721	Gastric triacylglycerol lipase	*Pinctada fucata*
CUST_51407	Unigene51446	Caffeoyl-CoA O-methyltransferase	*Pinctada fucata*
CUST_11562	Unigene11570	cytosolic glutathione S-transferase 1	*Oesophagostomum dentatum*
CUST_18671	Unigene18681	mu class glutathione S-transferase	*Crassostrea gigas*
CUST_25915	Unigene25932	glutathione S-transferase sigma class protein	*Crassostrea gigas*
CUST_28047	Unigene28066	glutathione S-transferase class mu	*Cyphoma gibbosum*
CUST_39791	Unigene39819	glutathione S-transferase	*Chironomus tentans*
CUST_54455	Unigene54495	glutathione S-transferase	*Branchiostoma belcheri tsingtauense*
CUST_56433	Unigene56473	microsomal glutathione S-transferase	*Venerupis philippinarum*
CUST_53080	Unigene53119	glutathione S-transferase pi	*Mytilus edulis*
CUST_53245	Unigene53284	microsomal glutathione S-transferase 1, isoform CRA_c	*Homo sapiens*
CUST_29733	Unigene29754	Temptin	*Haliotis discus discus*
CUST_12898	Unigene12906	Temptin	*Haliotis discus discus*
CUST_22403	Unigene22418	temptin	*Haliotis discus discus*
CUST_18929	Unigene18939	amiloride binding protein 1	*Danio rerio*
CUST_23905	Unigene23920	Semicarbazide-sensitive amine oxidase	*Equus caballus*
CUST_27372	Unigene27390	C. briggsae CBR-GST-11 protein	*Caenorhabditis briggsae*
CUST_36101	Unigene36123	ShTK domain containing protein	*Brugia malayi*
CUST_44222	Unigene44255	amiloride binding protein 1	*Bos taurus*
CUST_47036	Unigene47071	ovoperoxidase	*Lytechinus variegatus*
CUST_56279	Unigene56319	oxidase/peroxidase	*Aedes aegypti*
CUST_54925	Unigene54965	xanthine dehydrogenase/oxidase	*Tribolium castaneum*
CUST_54967	Unigene55007	melanogenic peroxidase	*Sepia officinalis*
CUST_56212	Unigene56252	Aspartate aminotransferase	*Bacillus stearothermophilus*

**Table 2:** **Expression profiles of biomineralization-related genes between two consecutive developmental stages identified in** ***Pinctada fucata***

**Probe name**	**Unigene name**	**Gene name**	**T/O FC**	**D/T FC**	**U/D FC**	**J/U FC**
CUST_24804	Unigene24820	Shematrin-4	-	-	-	2977.7↑
CUST_58195	Unigene58235	Pif	-	-	-	2116.9↑
CUST_38575	Unigene38603	KRMP 1	-	-	-	949.73↑
CUST_31	Unigene31	Shematrin-5	-	-	-	943.32↑
CUST_27219	Unigene27237	Pearlin	-	-	1.90	823.02↑
CUST_54654	Unigene54694	Tyrosinase	-	0.40↓	0.44↓	716.82↑
CUST_53622	Unigene53661	Prismalin-14	0.95	0.89	0.93	324.48↑
CUST_50742	Unigene50781	Shematrin-6	-	-	-	256.39↑
CUST_41072	Unigene41100	Tyrosinase-like protein 2	0.99	0.95	0.43↓	196.87↑
CUST_53862	Unigene53901	PFMG3	6.48↑	0.09↓	1.30	187.5↑
CUST_44067	Unigene44100	shell matrix protein	1.06	1.21	0.79	176.49↑
CUST_56052	Unigene56092	ACCBP 1	-	-	-	119.53↑
CUST_36021	Unigene36043	Prisilkin-39	0.77	1.21	0.84	119.48↑
CUST_31135	Unigene31156	MSI60-related protein	-	-	-	102.96↑
CUST_27662	Unigene27680	PFMG9	-	-	-	76.88↑
CUST_11152	Unigene11160	Tyrosinase-like protein 1	-	-	3.48↑	52.10↑
CUST_55983	Unigene56023	PFMG4	-	2.24↑	1.99	46.13↑
CUST_47372	Unigene47407	Shematrin-7	3.85↑	0.42↓	0.58	42.42↑
CUST_9246	Unigene9253	Calmodulin-like protein	-	-	0.79	40.16↑
CUST_57434	Unigene57474	Nacrein	1.54	1.24	0.63	8.38↑
CUST_367	Unigene367	molluscan prismatic and nacreous layer 88 kDa protein	-	-	-	5.06↑
CUST_50058	Unigene50096	PFMG6	-	2.03↑	5.82↑	4.53↑
CUST_1708	Unigene1709	PFMG11	-	-	-	2.94↑
CUST_45587	Unigene45621	PFMG8	0.40↓	2.1↑	13.42↑	2.66↑
CUST_24765	Unigene24781	Chitin synthase	3.39↑	75.81↑	0.83	1.34
CUST_45506	Unigene45540	PFMG1/7	0.85	0.84	0.92	1.63
CUST_40154	Unigene40182	PFMG2	0.41↓	5.25↑	1.15	1.56
CUST_53588	Unigene53627	N151	0.49↓	0.71	0.85	0.74
CUST_52216	Unigene52255	PFMG12	0.83	0.48↓	3.33↑	1.81
CUST_56226	Unigene56266	Ferritin-like protein	0.87	0.99	1.59	0.51

T/O FC, fold-change (FC) in gene expression comparing trochophore stage larvae with fertilized eggs by Significance Analysis of Microarrays analysis; D/T ratio, U/D ratio, J/U ratio are similar. ↑, up-regulated with FC ≥ 2, and ↓, down-regulated with FC ≤ 0.5.

### Expression of biomineralization-related genes during larval development

Although matrix proteins are important for regulating the biomineralization processes, such as pearl and shell formation in molluscs, the number of characterized matrix protein genes is limited [22]. Normal GO or KEGG term analyses do not perfectly match matrix proteins. Thus, we identified all genes involved in biomineralization in the oyster transcriptome, including the majority of known *P. fucata* matrix proteins like shematrin family, PFMG family, tyrosinase family and other proteins described before*,* and analyzed their expression profiles between two consecutive developmental stages [31-38]. Besides, the expression levels of other biomineralization-related proteins like calmodulin-like protein [35], molluscan prismatic and nacreous layer 88 kDa protein [38], N151 [39] and Ferritin-like protein [40] were also analyzed in Table 2. Interestingly, most of the characterized *P. fucata* matrix proteins were significantly up-regulated between the umbonal stage and juveniles, such as the shell matrix protein shematrin-4 (CUST_24804) at 2,977.7 FC. The majority of these genes were expressed at low levels in fertilized eggs to the umbonal stage but at high levels in juveniles, except for PFMG1/7 (CUST_45506), PFMG12 (CUST_52216) [31,34], and ferritin-like protein (CUST_56226). Several genes homologous to those related to biomineralization in other molluscan species were also investigated. Biomineralization-related genes, such as KRMP-7 (CUST_47674, *Pinctada margaritifera*), linkine (CUST_53921, *Pinctada margaritifera*) [41], Clp1 protein (CUST_56502, *Crassostrea gigas*), and Clp3 protein (CUST_57568, *C. gigas*) [42] were up-regulated in a similar pattern between the umbonal stage and juvenile stage (data not shown).

Chitin synthase (CUST_58227) and PFMG2 (CUST_40154) were highly expressed during the D-shaped stage. PFMG8, as well as PFMG6, were initially up-regulated at the D-shaped stage but then up-regulated significantly at the umbonal stage, suggesting potential roles regulating the formation of Prodissoconch II.

### Identifying the candidate genes involved in shell formation

Most known proteins related to shell formation exhibit an up-regulated expression level with > 20-fold changes from the umbonal stage to juveniles. But the functions of more genes with similar expression profiles remained unclear. We guess some of them might play roles in pearl or shell formation as well, which deserved to be further investigated. Based on this hypothesis, we investigated the up-regulated genes using some bioinformatics tools to detect potential matrix proteins. Among all 8,786 up-regulated genes between these two stages, 1,113 had a FC ≥ 20. Besides the known matrix proteins and genes related to biomineralization, as described above, 747 of these significantly up-regulated genes were not similar to any other known protein after annotation, whereas another 84 unigenes shared few similarities with some hypothetical or uncharacterized proteins without defined functions (Additional file 4).

As reported previously, 80% of the known matrix proteins encode secreted proteins and 74.6% contain tandem-arranged repeat units [22], suggesting some localized and modular features corresponding to their functions. These 831 genes were analyzed and divided into three classes. After excluding genes without an open reading frame (ORF) ≥ 80 aa, the remaining 138 genes were analyzed by a SignalP search, and 35 secreted proteins remained. A XSTREAM screen analysis was carried out, and five genes encoding secreted proteins also contained tandem-arranged repeat units. The FCs in these genes were: unigene18749_TP, 100.016-fold increase; unigene34354_TP, 22.37-fold increase; unigene35118_TP, 206.96-fold increase; unigene51738_TP, 21.94-fold increase; and unigene56675_TP, 152.68-fold increase. After a BLASTp search, unigene56675_TP shared 45% identity with nacre uncharacterized shell protein 5 (NUSP5, GenBank accession no: P86967) in *Pinctada margaritifera* [43], whereas the other four genes were not similar to any defined genes. As the functions of NUSP5 remain unclear, the roles of these candidate genes remain for further investigation. The nucleotide sequences and predicted coding proteins of the candidate genes are shown in Additional file 5.

### Expression patterns of the candidate genes in different tissues

**Figure 3:**
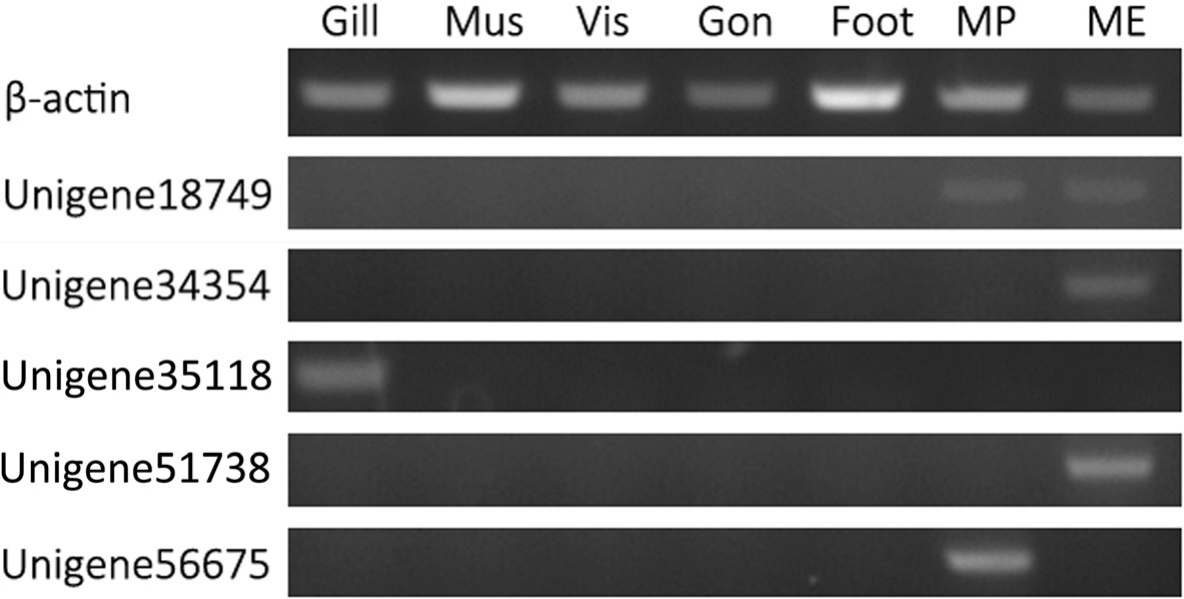
Expression patterns of the candidate genes in different *Pinctada fucata* tissues by semi-quantitative polymerase chain reaction analysis. Total RNA in adult oyster gill, adductor muscle (Mus), viscera (Vis), gonad (Gon), foot, mantle pallial (MP), and mantle edge (ME) was extracted and reverse-transcribed as templates. β-actin gene expression was used as a positive control.

The expression patterns of the five candidate genes possibly related to shell formation were detected in different tissues by semi-quantitative PCR. As expected, each of the genes exhibited tissue-specific expression profiles (Figure 3). Unigene56675 was expressed specifically in the mantle pallial, which secretes matrix proteins related to formation of the nacreous shell layer [6,8,14]. Unigene34354 and unigene51738 were expressed specifically in the prismatic layer-related tissue of the mantle edge [11,12,14]. In addition, unigene18749 was expressed both in the pallial and edge of the mantle, suggesting dual roles related to the nacreous and prismatic layers [7,24]. Unigene35118 was expressed at its highest in gill, but also exhibited a high expression level in the mantle edge according to a real time qPCR analysis (data not shown), probably corresponding to a predicted chitin binding domain in its coding protein. The role of unigene35118 remains to be further investigated. However, chitin is an important organic molecule related to biomineralization [44], particularly shell formation in molluscs [15,45-47], and some matrix proteins involved in forming the shell framework, such as prisilkin-39 [11], have chitin-binding ability.

### RNAi knockdown of the candidate genes

The RNAi knockdown approach has been used previously to investigate the regulatory roles of matrix proteins in the calcium carbonate crystallites during shell formation [48]. The microstructures of the nacreous and prismatic shell layers can be disrupted owing to down-regulated matrix proteins [8,23,24]. Because of this, the functions of the candidate genes were tested *in vivo* in RNAi experiments. An 80-μg aliquot of double-stranded RNA (dsRNA) was designed from each gene and injected into the adductor muscle of *P. fucata* adults with similar shell lengths. The efficiency of the RNAi experiment was confirmed 6 days later by real-time qPCR, resulting in 40–60% down-regulation of the corresponding candidate genes, with no significant influences on other matrix proteins like nacrein, pif and KRMP (Additional file 6).

**Figure 4:**
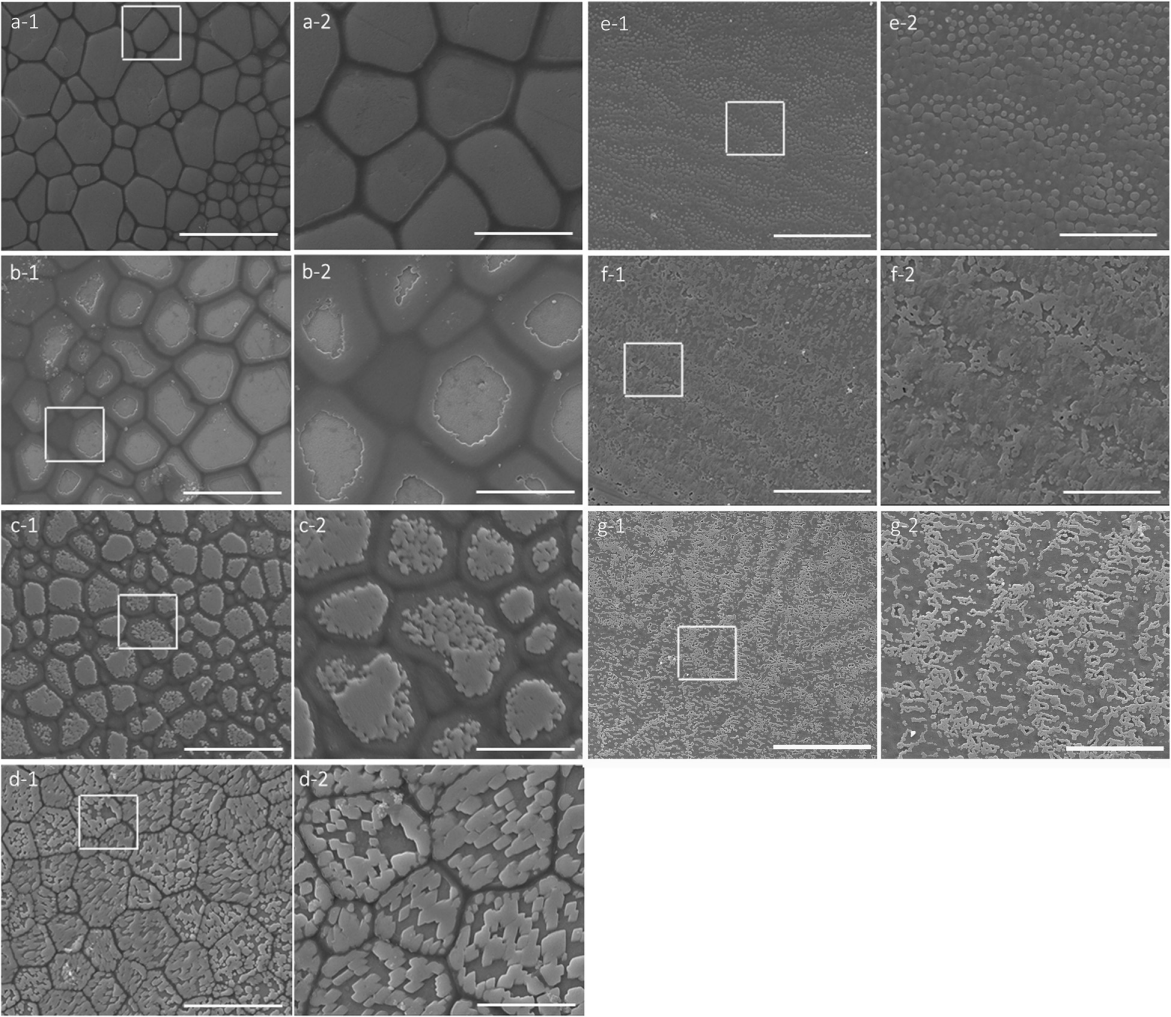
Effects of the candidate genes on regulating calcium carbonate crystallites on the shell surface. Expression levels of the candidate genes decreased following a dsRNA injection *in vivo*. **(a)** Scanning electron microscopic (SEM) images of a normal shell prismatic layer. **(b–d)** SEM images of the prismatic layers of RNAi knockdown group shells: unigene34354 **(b)**, unigene35118 **(c)**, and unigene51738 **(d). (e)** SEM images of a normal shell nacreous layer. **(f–g)** SEM images of the nacreous layers of RNAi knockdown group shells: unigene18749 **(f)** and unigene56675 **(g)**. a-2 shows an enlargement of the box in a-1, as do b-2–g-2. Bar, 50 μm in a-1, b-1, c-1, d-1, e-1, f-1, and g-1; bar = 20 μm in a-2, b-2, c-2, d-2, e-2, f-2, and g-2.

The inner surface structure of the shells was scanned by electron microscopy 6 days after the injection, and the shell nacreous and prismatic layers were observed separately (Figure 4). The nacreous and prismatic layers of the shells in the GFP-injected control group were normal, as seen in untreated oysters (Figure 4a, 4e). RNAi knockdown of Unigene34354 and unigene51738 would lead to disrupted phenomena in the prismatic layer. Knocking down of unigene51738 would lead to lacunose prismatic layer surface (Figure 4d) while inhibiting unigene34354 lead to an abnormal formation of the organic framework in prismatic layer (Figure 4b). Knocking down of the chitin-binding domain containing unigene35118 would lead to absence of the shell framework and lacunose prismatic layer surface (Figure 4c). These results indicate their potential roles in controlling the formation of prismatic layer.

On the other side, knocking down of Unigene18749 and Unigene56675 would both result in different and disrupted crystal deposition in the nacreous layer (Figure 4f, 4 g), suggesting their potential roles in controlling the formation of nacreous layer.

All of these data indicate a relationship between these candidate genes and shell formation, but their structures and functions during regulation of biomineralization remain to be further investigated.

## Discussion

Although genomics tools have begun to be used in marine bivalves in recent years [25-27], studies analyzing *P. fucata* genetic data remain insufficient [18,28,29,31,49]. Larval development investigations have focused mainly on tissue, organ, or shell developmental and structural observations [19,20,46,50], and knowledge about gene expression level changes is very limited [51-53], particularly during larval development [54]. The expression levels of only a few matrix proteins (nacrein, pearlin, MSI60, aspein, prismalin-14, and MSI31) at different larval development stages have been analyzed by RT-PCR and are related to shell formation in *P. fucata* [21]. Our present results have revealed global gene expression profiles during *P. fucata* larval development, particularly focusing on the relevance of the gene expression levels and the shell formation process. An analysis of differentially expressed genes across stage transitions would help reveal their potential roles in lots of biological processes including shell formation.

### Gene expression profiles and shell formation during larval development

The *P. fucata* developmental stages are the fertilized egg, trochophore, D-shaped stage, umbonal stage, juveniles, and adults. The calcium carbonate crystal polymorphism and the shell layer structure change throughout these stages [19,20]. At the early D-shaped stage, Prodissoconch I probably comprises ACC, whereas the aragonite and calcite Prodissoconch II appears during the umbonal stage. Finally, the dissoconch shell, with an inner aragonitic nacreous layer and an outer calcitic prismatic layer, forms at the juvenile stage [19,20,50]. Changes in matrix protein expression levels probably reflect their roles regulating shell formation.

**Figure 5:**
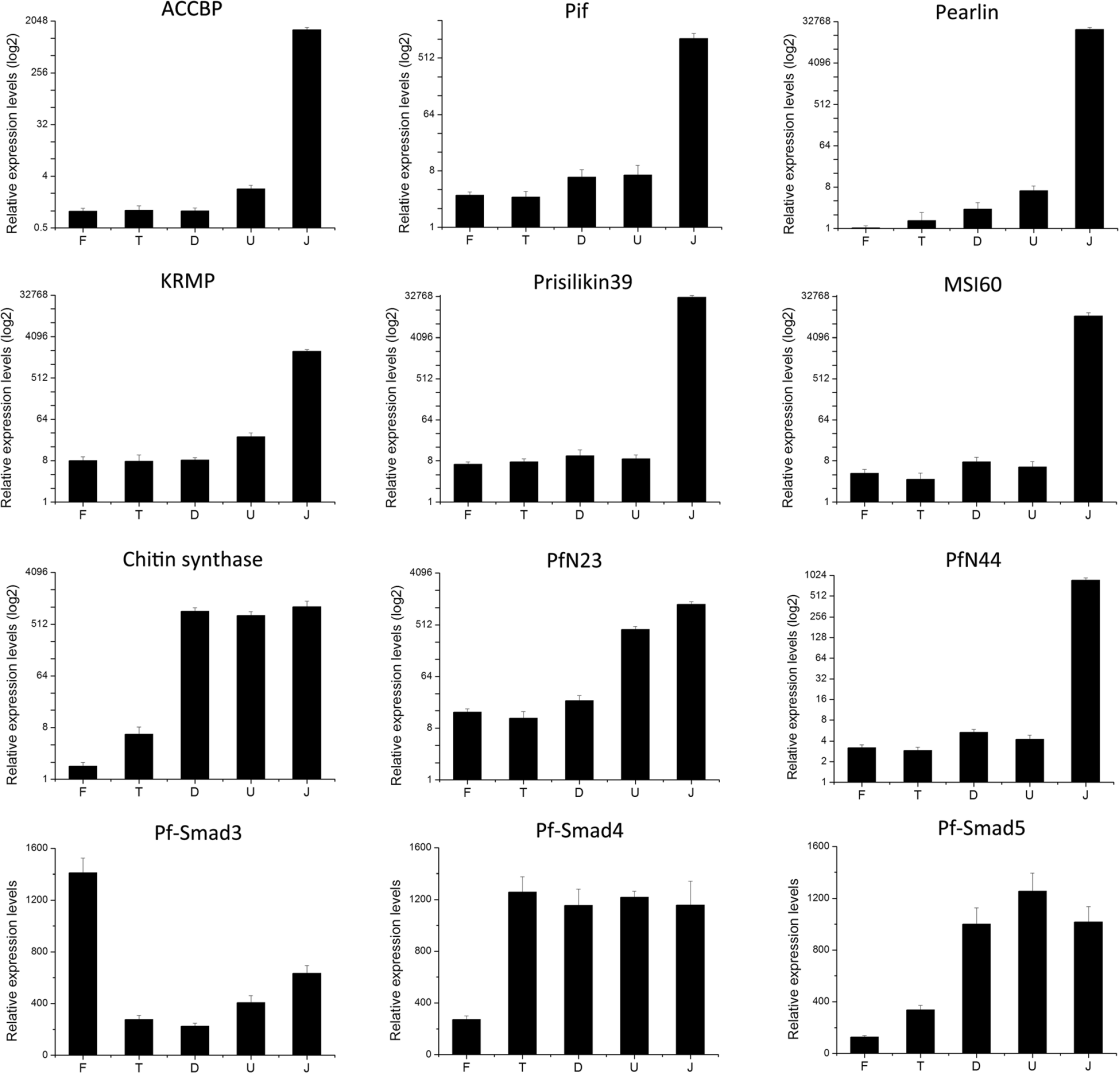
Expression patterns of 11 genes by reverse transcription-quantitative polymerase chain reaction analysis. Relative gene expression levels (log2) of matrix proteins ACCBP, KRMP-1, pearlin, Pif, prisilkin-39, MSI60, Chitin synthase, PfN23 and PfN44 were described before; while relative gene expression levels (linear) of transcription factors Pf-smad3, Pf-smad4, and Pf-smad5 exhibited different patterns. All of these results were positively correlated with the microarray data. F, fertilized eggs; T, trochophore stage larvae; D, D-shaped stage larvae; U, umbonal stage larvae; J, juvenile.

Chitin is an insoluble polysaccharide that comprises the body structure framework of molluscs and insects [47]. Chitinous material appears in the D-shaped stage and is widely distributed in larval shells of the oyster *Mytilus galloprovincialis*, where it presumably forms a chitin-protein complex [46]. Chitin synthase, which stimulates proliferation of mammalian chondrocytes by regulating synthesis of extracellular matrix components [42], is also an important matrix protein in the structural framework of shells [15,37,45]. In this study, chitin synthase expression was up-regulated significantly at the D-shaped stage and remained highly expressed since then (Table 2), which might be related to the synthesis of chitinous material of the larval shell, as well as other biological processes. Results of the RT-qPCR analysis revealed a similar expression pattern (Figure 5). The calcium signaling pathway was also predicted to be involved in this stage (Additional file 3), resulting from the large calcium requirement to form Prodissoconch I. Matrix protein PFMG2 is up-regulated at this stage, somehow indicating its potential role in the formation of Prodissoconch I, which need to be further investigated.

The majority of biomineralization-related genes were expressed at low levels during the umbonal stage, except for a few genes, such as PFMG6 and PFMG8, which were initially up-regulated during the D-shaped stage, and then were up-regulated to a greater extent at the umbonal stage. According to a previous study, knockdown of PfN23, disrupts the larval shell formation process at the early stage [22,23]. The microscopic structures of Prodissoconch I and Prodissoconch II differ from those of dissoconch shells [21], and expression of PFMG6, PFMG8, and PfN23 may significantly influence early formation of larval shells. In addition, a KEGG pathway analysis predicted that several amino acid metabolic pathways are active between the D-shaped and umbonal stages, which probably reflects the rapid larval growth at this time [19].

The majority of biomineralization-related genes were up-regulated at the juvenile stage with large FCs. These genes have crucial roles regulating formation of the nacreous and prismatic shell layers (Table 2). Besides the contigs listed, other contigs from the same biomineralization-related genes in the transcriptome exhibited similar expression patterns across stages, despite some fold-change value differences. Furthermore, gene expression profiles for chitin synthase, ACCBP, KRMP1, pearlin, Pif, prisilkin-39, MSI60, PfN23, PfN44 and three transcription factors, such as Pf-smad3, Pf-smad4, and Pf-smad5 (GenBank accession no: EU137731, KF307635, and KC462554) were also detected by RT-qPCR (Figure 5). This result confirms the positive correlation with microarray performance. Our data reveal that the highest expression level changes in most biomineralization-related genes corresponded to formation of the nacreous and prismatic shell layers, rather than construction of Prodissoconch I and Prodissoconch II.

In addition, the tyrosine metabolic pathway plays multiple roles in a wide array of biological processes, including pigmentation, innate immunity, wound healing, and sclerotization [55-58]. In particular, the final product is melanin but tyrosinase and other products from this metabolic process play important roles in cuticle sclerotization in insects [58,59]. In molluscs, the shell is the last barrier to the environment but serves a similar function to that of the insect sclerotized cuticle [31]. Tyrosinase, which is expressed particularly in the outer epithelial cells of the middle fold of the mantle, play roles in construction, pigmentation, and the periostracum shell covering [32,33]. In our study, the tyrosine metabolic pathway was continuously active from the D-shaped stage larvae to juveniles (Additional file 3), when the shell is initially constructed and gradually transformed into a typical bilayer structure. Among them, tyrosinase (CUST_54654, 716.82-fold increase), tyrosinase-like protein 1, (CUST_11152, 52.10-fold increase), and tyrosinase-like protein 2, (CUST_41072, 196.87-fold increase) were all up-regulated beginning in the larval umbonal through the juvenile stage (Table 2) and exhibiting a similar role as the majority of matrix proteins. Two homologous oxidase genes (CUST_47036 and CUST_56279) had a similar interesting expression pattern; they were up-regulated at the D-shaped stage (44.82- and 46.35-fold increases), down-regulated at the umbonal stage (223.99- and 204.60-fold decreases), and up-regulated again (219.42- and 112.70-fold increases) at the juvenile stage. These results suggest multiple functions of the tyrosine metabolic pathway during *P. fucata* larval development, including the formation of the periostracum of the shell, as well as other biological processes.

**Figure 6:**
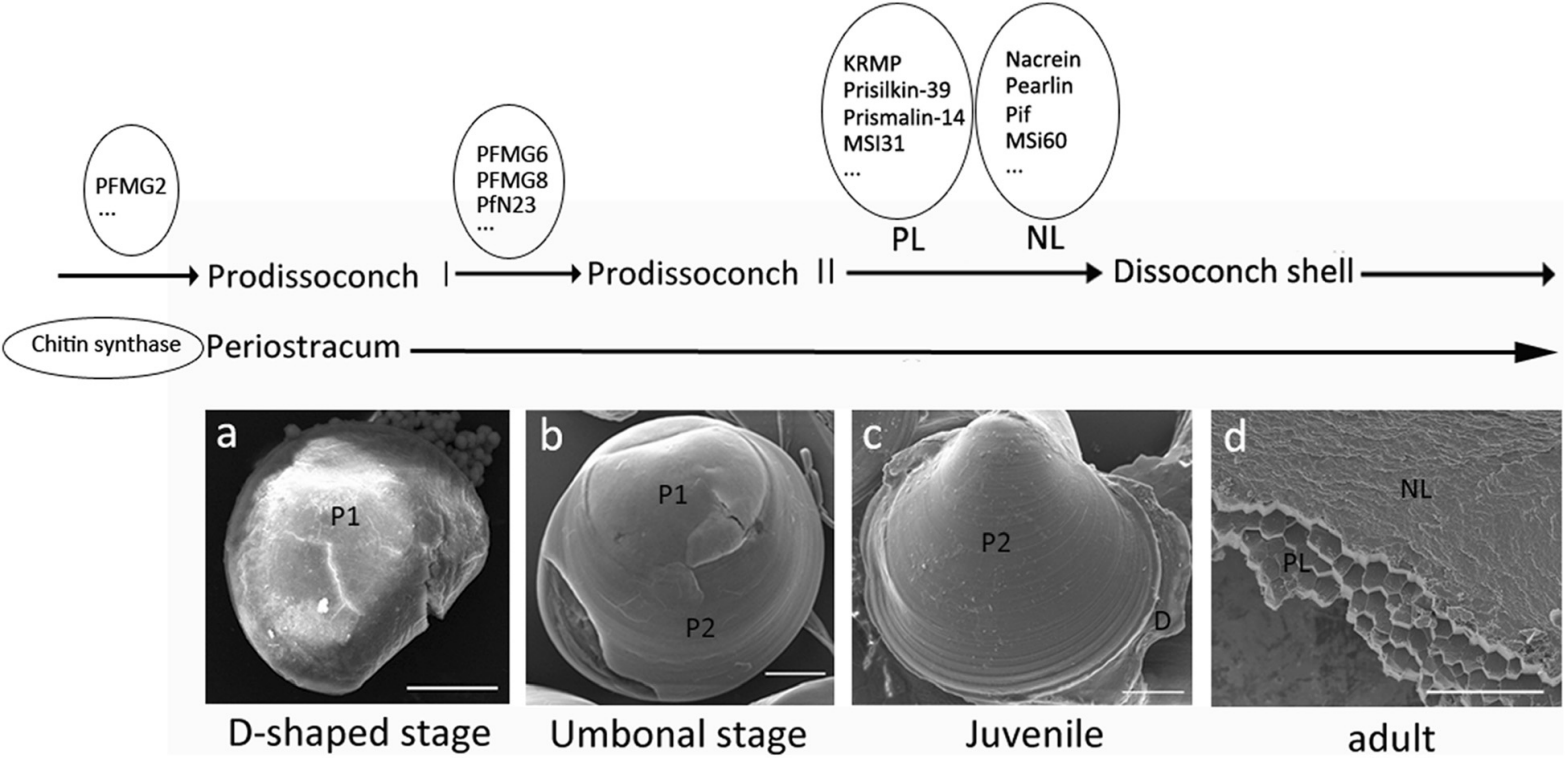
Expression patterns for the biomineralization-related genes regulating shell formation in *Pinctada fucata*. Bar, 20 μm in **a**, **b**, **c**; bar = 200 μm in **d**. P1, Prodissoconch I shell; P2, Prodissoconch II shell; D, Dissoconch shell; PL, prismatic layer; NL, nacreous layer.

These results accurately reflect the relationship between biomineralization-related genes and formation of prodissoconch or dissoconch shells; thus, demonstrating the importance of these genes regulating either the framework or the calcium carbonate crystallites during shell formation. We propose how different biomineralization-related genes regulate the larval shell formation process, based on expression patterns and potential functions (Figure 6).

### Identifying candidate genes involved in shell formation

Because of limited genetic information on *P. fucata*, 34,489 of the 58,623 unigenes in the transcriptome could not be annotated, which limits further investigation. As a result, identifying and characterizing additional genes involved in shell formation are important to help researchers better understand the regulatory mechanisms of bivalve biomineralization.

Identifying secreted proteins containing tandem-arranged repeat units has been recognized as a strategy for screening genes involved in *Haliotis asinina, Pinctada maxima,* and *P. fucata* shell formation [22,60]; using these methods, PfN23 and PfN44 have been determined to be involved in shell formation [23,24]. In this study, we analyzed the genes encoding secreted proteins and containing tandem-arranged repeat units from the over-regulated gene groups (FC ≥ 20) between the umbonal and juvenile stages, when the majority of biomineralization-related genes are highly up-regulated and the dissoconch shell is formed. We finally identified five new candidate genes probably involved in biomineralization. According to the semi-quantitative PCR analysis and RNAi knockdown experiment, these genes exhibited potential biomineralization-related roles both in their gene expression patterns and functions.

Unigene34354 and unigene51738 were expressed particularly in the mantle edge, which is related to the shell prismatic layer [11,12,14]. RNAi knockdown of these two genes would lead to disrupted phenomena in the prismatic layer. The calcitic prismatic layer surface became lacunose, whereas the organic framework remained clear after knockdown of unigene51738 (Figure 4d). However, inhibiting unigene34354 lead to an abnormal inner surface of the prismatic layer, which was probably caused by either activating growth of the organic framework or inhibiting deposition of calcitic prisms (Figure 4b). Unigene56675 is expressed mainly in the mantle pallial, which forms the shell nacreous layer [6,8,14]. RNAi knockdown of unigene56675 lead to disrupted crystal deposition in the nacreous layer (Figure 4 g). Unigene18749 was expressed both in the mantle pallial and mantle edge of the oyster, indicating a dual role regulating formation of the nacreous and prismatic layers. Injecting 80 μg dsRNA for knockdown lead to disordered morphology in the nacreous layer (Figure 4f), but no significant effect on the prismatic layer (data not shown).

However, the chitin-binding domain containing unigene35118 was expressed at its highest level in the gill, where chitin maintains filament shape [61]. However, its expression in the mantle edge remained higher than that in other tissues. Chitin is an important organic molecule during biomineralization [44], including shell formation in molluscs [15,45-47]. RNAi knockdown of unigene35118 lead to an abnormal prismatic layer, probably related to absence of the shell framework (Figure 4c). An investigation of this gene would probably shed new light on the mechanisms of chitin mineralization.

Taken together, our data indicate the potential of these genes to regulate shell formation.

## Conclusion

Lack of *P. fucata* genomic and larval development data limits further investigation into the regulatory mechanisms of biomineralization. Our gene expression profile analysis of the larval developmental stages was performed using a microarray platform. The expression levels of the biomineralization-related genes are regulated during larval development, probably corresponding to their function in larval shell formation, as well as other biological processes. For example, Chitin synthase and PFMG2 were up-regulated significantly beginning at the D-shaped stage, which might be related to synthesis of chitinous material or construction of the periostracum and Prodissoconch I. PFMG6, PFMG8, and PfN23 were initially up-regulated at the D-shaped stage and then were up-regulated significantly at the umbonal stage, indicating their potential roles regulating the formation Prodissoconch II, probably Prodissoconch I as well, which need to be further investigated. However, the majority of biomineralization-related genes are expressed at low levels early and then significantly up-regulated with large FCs at the juvenile stage, which might somehow indicate their crucial roles of these genes regulating formation of the nacreous and prismatic shell layers. The large variety of genes differentially expressed between developmental stages reveals the regulatory complexity of larval development, including shell formation. Five new genes, encoding secreted proteins containing tandem-arranged repeat units, exhibited similar up-regulated patterns at the juvenile stage. RNAi knockdown of these genes resulted in disrupted nacreous or prismatic shell layers, whereas four genes were expressed specifically in the mantle, reflecting their potential roles as matrix proteins. Our results bring a global perspective to the relationship between gene expression profiles and larval shell development in *P. fucata,* increase knowledge of biomineralization-related genes, and highlight new aspects of the shell formation mechanisms.

## Methods

### Larval culture

*Pinctada fucata* larvae were collected from the Daya Bay Marine Comprehensive Experimental Station, Shenzhen, Guangdong Province, China. The insemination and culture methods followed an earlier report [19]. Fertilized eggs were harvested immediately after insemination. The trochophore, D-shaped, and umbonal stage larvae, as well as the juvenile samples were collected 17 h, 48 h, 14 days, and 35 days after a microscopic count ensured that > 75% of the larvae had reached a particular growth stage.

### RNA extraction and cDNA synthesis

Total RNA was extracted from the larval samples and seven other tissues, including gill, adductor muscle, viscera, gonad, foot, mantle pallial, and mantle edge of adults using Trizol reagent (Invitrogen, Carlsbad, CA, USA) and a standard procedure. RNA was quantified at optical densities of 260/280 with an Utrospec 3000 UV-visible spectrophotometer (Amersham Biosciences, Uppsala, Sweden). RNA integrity was determined by fractionation on a 1.2% formaldehyde denatured agarose gel stained with Goldview.

Gene expression profiling during larval development was accomplished by DNA microarray using pooled samples from different stages (fertilized eggs, trochophore stage, D-shaped stage, and umbonal stage larvae, as well as juveniles). RNA was extracted as described above. Triplicate pooled samples were run for each stage point.

First-strand cDNA was produced for real time reverse transcription-quantitative polymerase chain reaction (RT-qPCR) using 1 μg total RNA from pooled samples of the different larval stages using PrimeScript™ RT Master Mix (Perfect Real Time; Takara Bio, Shiga, Japan). Tissue-specific semi-quantitative PCR was conducted using 1 μg total RNA of the different adult tissues to synthesize first-strand cDNA using MMLV-RT reverse transcriptase (Promega, Madison, WI, USA).

### *P. fucata* oligonucleotide microarray

Based on the adult mantle tissue transcriptome obtained from a previous study, 58,940 probes (60 nt), representing 58,623 transcripts, were synthesized *in situ*. In this study, total RNA of the different stages was separately reverse-transcribed and purified. The labeled cDNA samples were mixed and hybridized to the microarray, and the processed slides were scanned with an Agilent G2565CA Microarray Scanner (Agilent Technologies, Palo Alto, CA, USA).

### Statistical analyses

The scanned microarray images were analyzed using the Agilent Software Feature Extraction. GeneSpring GX software (CapitalBio, San Diego, CA, USA) was employed for quantile normalization and the statistical analysis. Cluster analyses were performed on the entire dataset using Cluster 3.0 software (CapitalBio). A principal components analysis (PCA) was used to determine the significantly differentially expressed genes between developmental stages. Expression profile comparisons were performed with the Significance Analysis of Microarrays (SAM) software [30]. Only genes whose changes were consistent (p-value < 0.05, fold-change [FC] ≥ 2) were selected as differentially expressed genes. A scatter plot was also prepared. The non-parametric Spearman’s rank-correlation test was used to assess the correlation between the expression values measured by real time RT-PCR and microarray for the set of candidate genes, using SPSS 12.0 software (SPSS, Inc., Chicago, IL, USA). The functional enrichment of up- or down-regulated genes during two consecutive developmental stages was assessed based on the GO [62] and KEGG [63] pathways annotation terms. Only the annotations with a q-value < 0.05 were considered significant.

### Candidate biomineralization-related gene screening and bioinformatics

Among all unigenes up-regulated from the umbonal to the juvenile stage, 1,113 unigenes increased expression > 20-fold and were further analyzed to screen for candidate biomineralization-related genes contributing to larval shell formation. A total of 831 of these unigenes were not similar to any known protein or they had some similarities between hypothetical or uncharacterized proteins after a BLASTX search using the GenBank nr database.

All of these unigenes were considered uncharacterized and the nucleotide (5′-3′) and amino acid sequences of the coding regions were investigated using ESTScan [64]. The following bioinformatics tools were employed to search for secreted proteins [65]. Unigenes with a coding region < 240 base pairs were discarded, and the remaining potential coding regions were searched for signal peptides using SignalP v4.1 [66]. Peptide-positive proteins targeted to organelles were removed after a TargetP [67] search, and transmembrane proteins were removed using the TMHMM Service v. 2.0 (http://www.cbs.dtu.dk/services/TMHMM/). XSTREAM [68] was used to isolate proteins with tandem-arranged repeat units using the default settings. The known *P. fucata* matrix proteins were checked as a control.

### Real-time qPCR

A set of 11 genes was tested by RT-qPCR using the same samples used in the microarray experiments to validate the microarray platform results. The genes were chosen from those displaying different expression patterns during larval development. Fifteen samples from the five pools were employed. PrimeScript™ RT Master Mix (Perfect Real Time) (Takara) was used to perform the RT-PCR experiments, following the manufacturer’s instructions in a *LightCycle 480* thermocycler (Roche Diagnostics, Mannheim, Germany). The 18S RNA gene was chosen as the negative control [21], as its expression did not change significantly during larval development. All primers used in this study are shown in Additional file 7.

### Tissue specificity analysis by semi-quantitative PCR

According to previous studies, most matrix proteins were specifically expressed in mantle tissue; and the different regions of mantle pallial or edge referred to the formation of either nacreous or prismatic layer of the shell. As a result, a tissue specificity analysis by semi-quantitative PCR would help identify the candidate genes involved in shell or pearl formation. Five pairs of primers corresponding to the candidate genes were designed to analyze the tissue expression patterns in the pearl oyster, and β-actin was used as the positive control. Synthesized cDNA from gill, adductor muscle, viscera, gonad, foot, mantle pallial, and the mantle edge of adult oysters was used as the template for the PCR. All PCR products were sequenced and confirmed.

### RNAi experiments

RNAi was performed following methods described previously [8,22]. The primers were designed to amplify specific sequences. dsRNA was synthesized and purified using the RiboMAXTM Large Scale RNA Production System (T7) kit (Promega) *in vitro*. Template DNA were digested with RNase free DNase I (Takara). dsRNA was diluted to 80 μg/100 μL in PBS, and 100 μL was injected into the adductor muscle of adult oysters. The same quantity of green fluorescent protein (GFP) dsRNA synthesized from pEGFP-C (Promega) and 100 μL PBS were used as controls.

Total RNA was extracted from the mantle tissue of each oyster 6 days after the injection to synthesize first-strand cDNA as described above. Real time RT-PCR was conducted to investigate RNAi efficiency. The shells were washed with Mili-Q water, cut into pieces, and air-dried. The nacreous and prismatic layers were scanned with a FEI Quanta 200 scanning electron microscope.

### Availability of supporting data

Microarray data supporting the results of this article are available in the NCBI Gene Expression Omnibus datasets (GEO, http://www.ncbi.nlm.nih.gov/gds/) in the Bioproject PRJNA269165 under Accession Number GSE63824.

## Additional files

Additional file 1:
**Heat map of contigs differentially expressed between the 15 pools from the five developmental stage samples.** The three biological replicate pools at each developmental stage shared a similar expression pattern, whereas samples at different stages exhibited unique more or less expressed genes. The normalized expression levels are depicted with a color scale, in which shades of red represent higher expression and green represents lower expression. Additional file 2:
**List of Cellular Component Gene Ontology (GO) terms enriched between two consecutive developmental stages.** Only terms with a q-value < 0.05 were considered to be “true” (XLS). Additional file 3:
**List of Encyclopedia of Genes and Genomes (KEGG) pathway components enriched between two consecutive developmental stages.** Only terms with q-value < 0.05 were considered to be “true” (XLS). Additional file 4:
**List of contigs significantly up-regulated between the umbonal larval stage and juveniles.** A total of 282 “annotated” contigs and 831 “characterized” contigs were evaluated (XLS). Additional file 5:
**The nucleotide sequences and predicted open reading frames (ORFs) of the five candidate shell formation genes.** The yellow shadow refers to the predicted signal peptide and the underlined amino acid sequences of unigene35118 show the predicted chitin binding domain (PDF). Additional file 6:
**Expression levels of selected genes knocked down by RNAi.** The expression levels of the selected genes were measured by real-time quantitative PCR (PDF). Additional file 7:
**Primer sequences designed for this study (PDF).**
