# Supplementary figures and images for: Microarray: a global analysis of biomineralization-related gene expression profiles during larval development in the pearl oyster, Pinctada fucata
Source: BMC Genomics. 2015 Apr 19;16(1):325. doi: 10.1186/s12864-015-1524-2 (PMC4445274; doi:10.1186/s12864-015-1524-2)

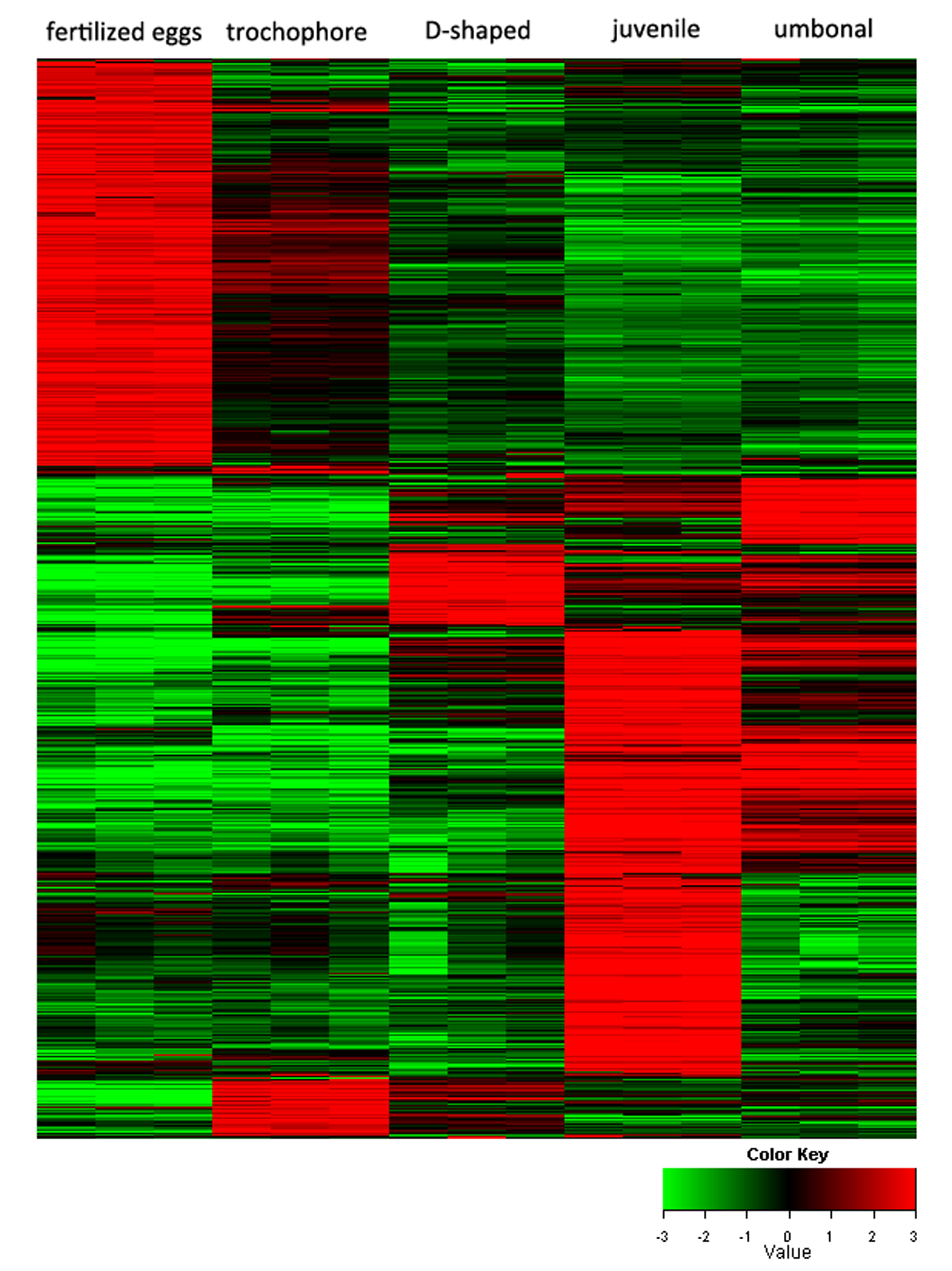

Supplement: Additional file 1: — Heat map of contigs differentially expressed between the 15 pools from the five developmental stage samples. The three biological replicate pools at each developmental stage shared a similar expression pattern, whereas samples at different stages exhibited unique more or less expressed genes. The normalized expression levels are depicted with a color scale, in which shades of red represent higher expression and green represents lower expression. [file 12864_2015_1524_MOESM1_ESM.tiff]
