# Supplementary material for: Microarray: a global analysis of biomineralization-related gene expression profiles during larval development in the pearl oyster, Pinctada fucata
Source: BMC Genomics. 2015 Apr 19;16(1):325. doi: 10.1186/s12864-015-1524-2 (PMC4445274; doi:10.1186/s12864-015-1524-2)
Supplement: Additional file 5: — The nucleotide sequences and predicted open reading frames (ORFs) of the five candidate shell formation genes. The yellow shadow refers to the predicted signal peptide and the underlined amino acid sequences of unigene35118 show the predicted chitin binding domain (PDF). [file 12864_2015_1524_MOESM5_ESM.pdf]

1. **Unigene18749**

Nucleotide sequence:

CGGAATAACATTACTGAGACTTTTAGAAAATCAGGGAAAAACATTATATAACAGCGGGATATTA
TATCCAAGATAATTAAGAAACATGAAACGGATTTATGTTCTAGTTCTCTTATTCATTTTGCTTGTG
TGTATAGCGGAGGCGCAAAAGAAATCCAAAGATAGCAAAAAAGCATCAAGCAAATCCTCAAG
CAAATCCTCTGGCAAGTCTAAATCGTCTCCAAAGTCATCCGGGGCCAAAGGCAAGTCTCCTAC
CCCATCGGCCCCGGATGACTTTGGAGACGATTTAGACTTGCCAGAGGATTTGCTTGAGGATTT
GCTTGATGCTTTTTTGGTCAAACAGACTACTCAAAGCTATCAAGGCCGGGGAACAAATGCCAC
CACCGATGTGTCCGAATGGTCTACCAAAAGCTGACTGTTCTCCAATTGCATGTGACAAATGGA
CATGTTCTAATATATTGAACACTGTATGCAAGGAACAGTGTCACGTTTGCGAGCCTAAATTCTA
CATTGGTGGAAGTGAAGTTACTCAATTCTGTGAATTAAAACCTGCCAACACGCAGCCTCGGGC
AAATCAGTCTCCTCCAACATCACGTAATACGGCAACAGATCAAGGATCACAGAATTCGGGACC
TAGCTCCAATGGGGCACCATCAAATATGCCGCCCATGCCGGGCATGCCAATGATGTTTTCTGA
GAACCCAATGCCAATGGGCGGACCACCCGGAATGGAATTTATGCCAAACTTTGAAAATTTCCC
TCCTGGAATGAGTCCTATGCAATTTTTCCATCATCTACAGAATATGAACATGCCGAATGAAAAC
CAGGGAATAAGATCACAGGCCAATTAAAATATTTCCTTGTTCAGCGTTTGATTTTCATCAACAA
TTTTATTTTCAGTTTTCATCATCACCATATTTTGTTGAAATTTTTAATAACTTTGCATTAACCATAC
ATATTTTACTGCCA

PREDICTED ORF:

MKRIYVLVLLFILLVCIAEAQKKSKDSKKASSKSSSKSSGKSKSSPKSSGAKGKSPTPSAPDDFGDDL
DLPEDLLEDLLDAFLVKQTTQSYQGRGTNATTDVSEWSTKS

2. **Unigene34354_TP**

Nucleotide sequence:

TCAATATTCTGTCTTTTCAGAACACAGGAAGCATACTCAAGCATGAAATTACAGACTTCATTAG
TCATCATGTGTGTTCTCTGCACCATCTGTTATACAGCCCCTTGGAATTTGGAGGATCTTAACCTT
GAAAACGCGATGGATGAAGAAAATGACGATTTCGAGAAGAATAGACGAATGGTCAATGGAG
ACAGTAGTAGAAGCAACAGTGAGGGATTCAGTTCTGTATTGGAAAGGAGAAATCTAATTGCT
GCTATTGCTGCTGCTCAAAGCCAACAAGCTTTAAACAAGTTGATGGCGCAAATGATGAATCAA
ATGATGAACCAAATGACAAATTCTCAAAGGCAAAGAATTCGGCAACAGATGAGTCANNNNN
NNNNNNNNNNNNNNNNNNNNNNNNNNNNNNNNNNNNNNNNNNNNNNNNNNNNNNNNN
NNNNNNNNTTTTGGATTTAATTAGATTAACCTCGTAATCAGAACTCTGCCAACACATCTTAAA
CTGCGA

PREDICTED ORF:

MKLQTSLVIMCVLCTICYTAPWNLEDLNLENAMDEENDDFEKNRRMVNGDSSRSNSEGFSSVL
ERRNLIAAIAAAQSQQALNKLMAQMMNQMMNQMTNSQRQRIRQQMSXXXXXXXXXXXX
XXXXXXXXXXFGFN

3. **Unigene35118_TP**

Nucleotide sequence:

CGGAAAGAAAGCGAAATAGTAGACTGGAAAATGATGGAAAAATTGATATTTTTCTGTTTTTTG
ATGCCGTTAATGTGGAATCATGTCTCTTCACATGGTAGATTAATAAGACCACCATCCCGAAATTC
AGCATGGCGATATCATCTCAGTGATGTACAGGATTACAACGATATGGGACACAATTGTGGAGG

AAAAAATTATCAATGGTCGAATGGAGGAAAATGTGGATTATGCGGAGATCCCTTTGATGGTGT
ACAGGATAACATGGCTGGTGGAAAATACGGTAAGGCTACCAGAAATATCACAGGATGCTACG
AATTGGCGTCACCAACAGAAATAGATGTGGCGGTACAGATCACAGCCCAGCATAAGGGATTC
TTTGAATTCAGACTTTGCGTGAACAATAATTTGAGCAAACCCATTACACAAGAATGCCTTGATG
AACACCTGCTGAAAATCAATGGTACAGACGAGACTCGATTCTATGGGATATCAGAAAACAAAG
TTTACAATCTAAAACTAAGTGTGCCAGGAAACATTCAGTGTGACCAATGCGTCCTACAATGGA
AATGGAATACGGGAAACTCCTATGGCTGCATTCCGGGGACGAATGATTGTGGAATTGGCTTTG
GCCAACAAGAACAGTTTTATGCTTGTGCCGATATTTCTATCAAAGGAAACTGCAATAGAGGTG
GAACTACGATTTTGCCGACTCAAGCGCCTCTTACAGAAGCACCTTGGTATCCAGCACCAATGA
CAGCGGCACCACAGACGCAAGCACCAATGACAGCGGCACCACAGACACAAGCGCCAATGAC
AGCGGCACCACAGACGCAAGC

PREDICTED ORF:

MMEKLIFFCFLMPLMWNHVSSHGRLIRPPSRNSAWRYHLSDVQDYNDMGHNCGGKNYQWS
NGGKCGLCGDPFDGVQDNMAGGKYGKATRNITGCYELASPTEIDVAVQITAQHKGFFEFRLCVN
NNLSKPITQECLDEHLLKINGTDETRFYGISENKVYNLKLSVPGNIQCDQCVLQWKWNTGNSYG
CIPGTNDCGIGFGQQEQFYACADISIKGNCNRGGTTILPTQAPLTEAPWYPAPMTAAPQTQAPM
TAAPQTQAPMTAAPQTQ

4. **Unigene51738_TP**

Nucleotide sequence:

TACGATTCCCTGTGAAAGACAATCAAACACCAGTGATATCCACCGAGATGAAGTCAGCTACGG
TAGCAGTCCTATTTGCCGTGTTATTCACAGCGTACGGAAATCCATGGAATTATCCAGGAAGATA
TTATTATGGATACTCCAATTGCTATGACTATGACTGTAATGGAATGAACTGCAGATCTATGGGTA
ACGACTTTTATTGTATGACAAACTATAGATATTACCCCATGTTTGGTAACCATCACTCACGATCAT
GTGAATGCAGGAACATGAACATGGGTAGAGAGTTCTTCTATCGTAACAACTGGAACAACTATA
ACATGAACAATTGGTGGAACAGATGGAACAGGAACAACTGGTGGAATAACTGGAAC

PREDICTED ORF:

MKSATVAVLFAVLFTAYGNPWNYPGRYYYGYSNCYDYDCNGMNCRSMGNDFYCMTNYRYYP
MFGNHHSRSCECRNMNMGREFFYRNNWNNYNMNNWWNRWNRNNWWNNWN

5. **Unigene56675_TP**

Nucleotide sequence:

GTGGATGTACGTGCTCAGAATGAGATAATAACAGTTTCAAAATGAGCAGGCTAACGTTACTGG
CGTTATTGGTTGCTGTGGTAGTTATTTCACGTGTGCTAGGTCAAGGCCAAGAGAAAGAAAAA
AGGAACGAACATGAACCAGGAAACCAGGATGGTAGAAATCAAAATGAAAGAAGAGAAAGA
AATTTGAAAATACCAACACGAAAGAGCCGTCGGATAGTAAACAGAAATAATAACGGGAAACA
GATGCAAACTTGGCAACGTCAAAACTATGTCAGGAAATACCGCCGCATTTCTCCTACGAACAC
TATTAATCGTAACAACGGATCAAAAGGCAAGTCACAAAATGGTGGCAGCGGTGGGAATGGCT
CCGGAAATAATGTACAAGAATGGAAAAAGACGACACTACCAATAGCAAAACGCTTCCGAAAT
AGAAATTCATCGAACGCACCTAGAAACGGCGGCAATATGAAACAGCAGCAATATAGTAGACG
GAACAGGAGATGGAATGGAATGAATAGAAGAGCCCGTCCTACAAGACGGTACTACCCACGTG
TATATGGAAGATATTACCAATATGGTCCATATATATGGTACAGATATGGGAACAAATGGAAGTTT
GTCGGATACAACAGAATGTACGGAAGGTACAGAAGGCCCACAAAAACCAAGAAGGCAACTC
AGAATAACCAAGGAGA

PREDICTED ORF:

MSRLTLLALLVAVVVISRVLGQGQEKEKRNEHEPGNQDGRNQNERRERNLKIPTRKSRRIVNRN
NNGKQMQTWQRQNYVRKYRRISPTNTINRNNGSKGKSQNGGSGGNGSGNNVQEWKKTTLP
IAKRFRNRNSSNAPRNGGNMKQQQYSRRNRRWNGMNRRARPTRRYYPRVYGRYYQYGPYIW
YRYGNKWKFVGYNRMYGRYRRPTKTKKATQNNQG
