# Supplementary material for: Microarray: a global analysis of biomineralization-related gene expression profiles during larval development in the pearl oyster, Pinctada fucata
Source: BMC Genomics. 2015 Apr 19;16(1):325. doi: 10.1186/s12864-015-1524-2 (PMC4445274; doi:10.1186/s12864-015-1524-2)

a) 80 μg dsRNA of Unigene18749 injected

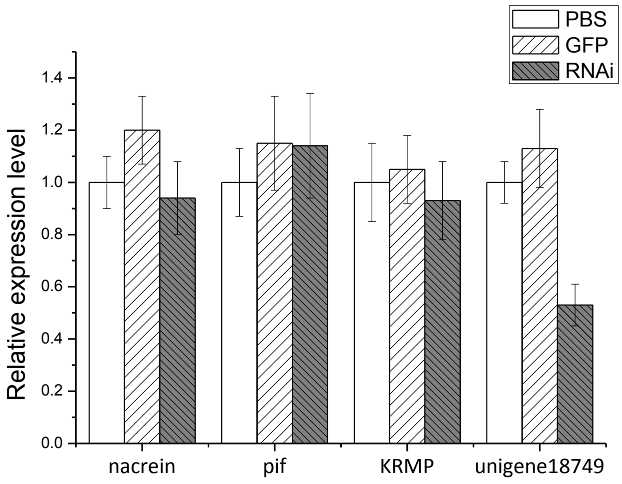

b) 80 μg dsRNA of Unigene34354 injected

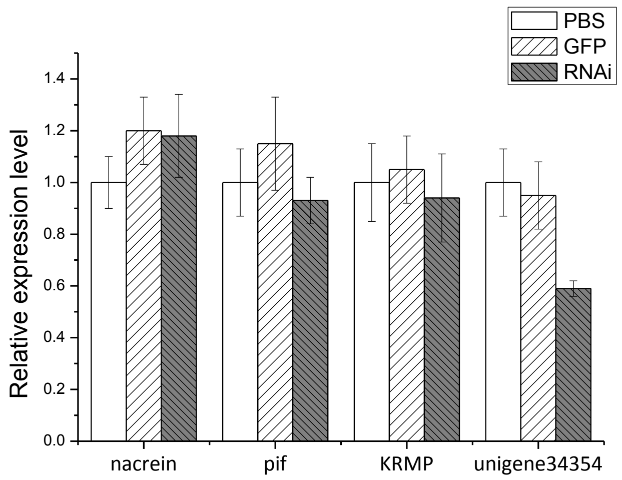

c) 80 μg dsRNA of Unigene35118 injected

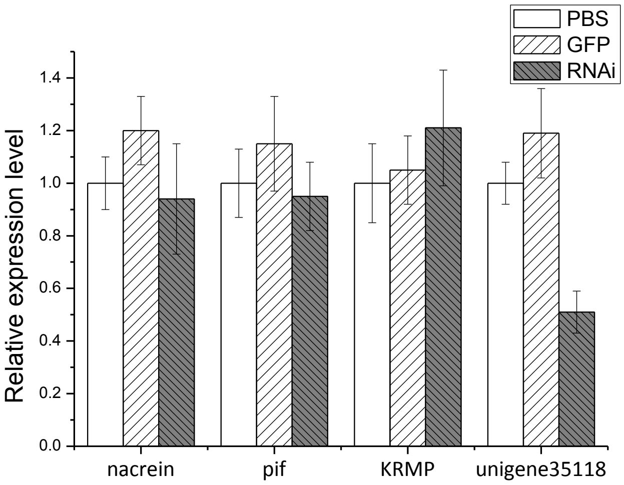

d) 80 μg dsRNA of Unigene51738 injected

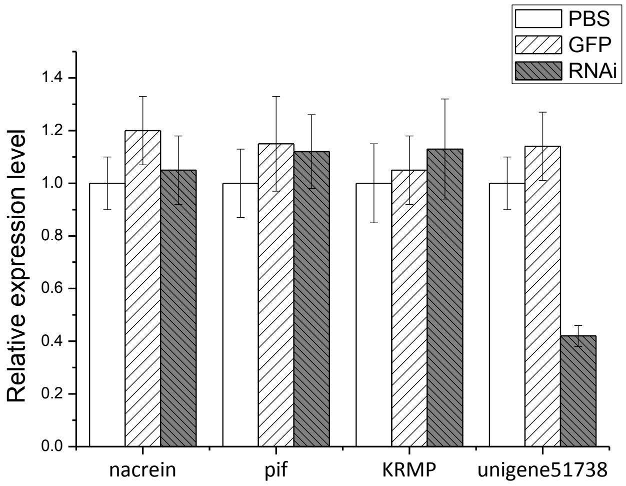

e) 80 μg dsRNA of Unigene56675 injected

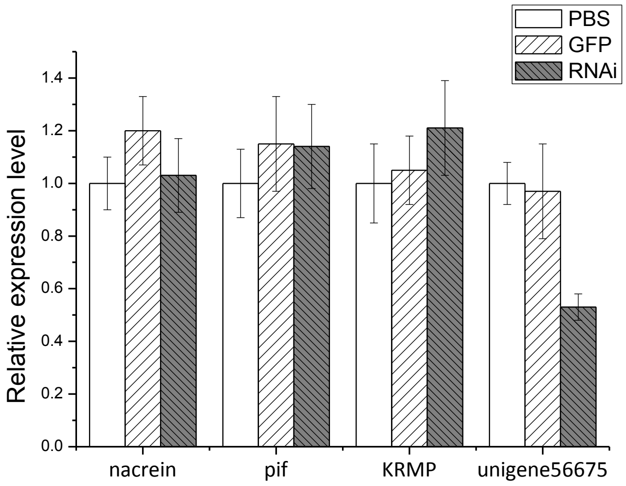

Supplement: Additional file 6: — Expression levels of selected genes knocked down by RNAi. The expression levels of the selected genes were measured by real-time quantitative PCR (PDF). [file 12864_2015_1524_MOESM6_ESM.pdf]
