# Supplementary material for: Microarray: a global analysis of biomineralization-related gene expression profiles during larval development in the pearl oyster, Pinctada fucata
Source: BMC Genomics. 2015 Apr 19;16(1):325. doi: 10.1186/s12864-015-1524-2 (PMC4445274; doi:10.1186/s12864-015-1524-2)
Supplement: Additional file 7: — Primer sequences designed for this study (PDF). [file 12864_2015_1524_MOESM7_ESM.pdf]

Additional file 1: Table S1. Designed primers sequences used in this study.

Primer name	Primer sequences (5' to 3')
**1. semi-quantitative PCR**	
actin-F	CTCCTCACTGAAGCCCCCCTCA
actin-R	ATGGCTGGAATAGGGATTCTGG
18749-F	GCGAAATGCAGAAACTAGCAG
18749-R	TAGCTGATGTTGGAGGAGACC
34354-F	GTGTTCTCTGCACCATCTGTTA
34354-R	CTGACTCATCTGTTGCCGAAT
35118-F	CCTTGATGAACACCTGCTGAA
35118-R	AATCGTAGTTCCACCTCTATTGC
51738-F	TCACGATCATGTGAATGCAGGA
51738-R	GTTCCAGTTATTCCACCAGTTGTTC
56675-F	AAGACGACACTACCAATAGCAA
56675-R	TACCTTCCGTACATTCTGTTGT
**2. RNAi experiment**	
GFP-dsF	GCGTAATACGACTCACTATAGGGAGAATGGTGAGCAAGGGCGAGGAG
GFP-dsR	GCGTAATACGACTCACTATAGGGAGATTACTTGTACAGCTCGTCCATG
18749-dsF	GCGTAATACGACTCACTATAGGGAGAGCGAAATGCAGAAACTAGCAG
18749-dsR	GCGTAATACGACTCACTATAGGGAGATACGTGATGTTGGAGGAGACC
34354-dsF	GCGTAATACGACTCACTATAGGGAGAACGAATGGTCAATGGAGACAG
34354-dsR	GCGTAATACGACTCACTATAGGGAGACTGACTCATCTGTTGCCGAAT
35118-dsF	GCGTAATACGACTCACTATAGGGAGAACAGGATGCTACGAATTGGC
35118-dsR	GCGTAATACGACTCACTATAGGGAGACCCGTATTCCATTTCCATTG
51738-dsF	GCGTAATACGACTCACTATAGGGAGAGCAGTCCTATTTGCCGTGTTA
51738-dsR	GCGTAATACGACTCACTATAGGGAGATGTTCCTGTTCCATCTGTTCC
56675-dsF	GCGTAATACGACTCACTATAGGGAGAAGAAATTCATCGAACGCACC
56675-dsR	GCGTAATACGACTCACTATAGGGAGATTTTGTGGGCCTTCTGTACC
**3. Real time quantitative PCR**	
RT-18S -F	GGGAATCAGGGTTCGATTCC
RT-18S -R	CCTGCTGCCTTCCTTGGA
RT-Pif-F	TGCTGCCATCACGTGAGTATG
RT-Pif-R	GACTTCCCTTTCTCACACTTCCA
RT-ACCBP-F	GACATGGAACAAAGATGGTGGA
RT-ACCBP-R	CTGTGGCTGGAATGGTTGG
RT-P39-F	CTGGAATGAGAGGATATG
RT-P39-R	TGCTGCTGTAATAACTATA
RT-Pearlin-F	TGCGGACGTTACTCATACTGCT
RT-Pearlin-R	CCACATCTAAGCCACTCATACCTCT
RT-MSI60-F	AGGTGGACGAAGAGGTAGAGGTAG
RT-MSI60-R	AGCGGCAGCGGCAACATC
RT-KRMP1-F	TTTCCTCCTCCTTGGAGCATTC
RT-KRMP1-R	CCAAAGTTGTAATCATCGCCACC
RT-Tyrosinase-F	AGGAGGGATGCCATTTGATA

RT-Tyrosinase-R	TGGAATGAATTATGGTGTTTGG
RT-Chitin syn-F	CCAAATTGCCAACCATCTG
RT-Chitin syn-R	GAAAGACTCTCCCTCAGACTATGG
RT Smad5-F	ATGGGCGGAGAAAGCCGTAGA
RT Smad5-R	GCCCTTACGATGGGACACTTG
RT Smad3-F	TATTGAAGCACCAGTGTTGCCTCC
RT Smad3-R	TCTAAAGGTGGGCTTGGTGAAACG
RT Smad4-F	CCTGGATAGGTTCTGTTTGGG
RT Smad4-R	TCTGACGGTGACACTGACGAA
